# Somatic Genomics and Clinical Features of Lung Adenocarcinoma: A Retrospective Study

**DOI:** 10.1371/journal.pmed.1002162

**Published:** 2016-12-06

**Authors:** Jianxin Shi, Xing Hua, Bin Zhu, Sarangan Ravichandran, Mingyi Wang, Cu Nguyen, Seth A. Brodie, Alessandro Palleschi, Marco Alloisio, Gianluca Pariscenti, Kristine Jones, Weiyin Zhou, Aaron J. Bouk, Joseph Boland, Belynda Hicks, Adam Risch, Hunter Bennett, Brian T. Luke, Lei Song, Jubao Duan, Pengyuan Liu, Takashi Kohno, Qingrong Chen, Daoud Meerzaman, Crystal Marconett, Ite Laird-Offringa, Ian Mills, Neil E. Caporaso, Mitchell H. Gail, Angela C. Pesatori, Dario Consonni, Pier Alberto Bertazzi, Stephen J. Chanock, Maria Teresa Landi

**Affiliations:** 1 Division of Cancer Epidemiology and Genetics, National Cancer Institute, Bethesda, Maryland, United States of America; 2 Advanced Biomedical Computing Center, Frederick National Laboratory for Cancer Research, Leidos Biomedical Research Inc., Frederick, Maryland, United States of America; 3 Cancer Genomics Research Laboratory, Frederick National Laboratory for Cancer Research, Leidos Biomedical Research Inc., Frederick, Maryland, United States of America; 4 Center for Biomedical Informatics and Information Technology, National Cancer Institute, Bethesda, Maryland, United States of America; 5 Division of Thoracic Surgery, Fondazione IRCCS Ca’ Granda—Ospedale Maggiore Policlinico, Milan, Italy; 6 Division of Thoracic Surgery, Istituto Clinico Humanitas, Rozzano, Milan, Italy; 7 Thoracic Surgery Unit, Community Hospital, Brescia, Italy; 8 Information Management Services, Inc., Rockville, Maryland, United States of America; 9 Center for Psychiatric Genetics, Department of Psychiatry and Behavioral Sciences, North Shore University Health System Research Institute, University of Chicago Pritzker School of Medicine, Evanston, Illinois, United States of America; 10 Department of Physiology & Cancer Center, Medical College of Wisconsin, Milwaukee, Wisconsin, United States of America; 11 Division of Genome Biology, National Cancer Center Research Institute, Tokyo, Japan; 12 Departments of Surgery and of Biochemistry and Molecular Biology, Norris Comprehensive Cancer Center, Keck School of Medicine, University of Southern California, Los Angeles, California, United States of America; 13 Prostate Cancer UK/Movember Centre of Excellence for Prostate Cancer Research, Centre for Cancer Research and Cell Biology, Queen’s University, Belfast, United Kingdom; 14 Epidemiology Unit, Fondazione IRCCS Ca’ Granda—Ospedale Maggiore Policlinico, Milan, Italy; 15 Department of Clinical Sciences and Community Health, Universita’ degli Studi di Milano, Milan, Italy; MSKCC, UNITED STATES

## Abstract

**Background:**

Lung adenocarcinoma (LUAD) is the most common histologic subtype of lung cancer and has a high risk of distant metastasis at every disease stage. We aimed to characterize the genomic landscape of LUAD and identify mutation signatures associated with tumor progression.

**Methods and Findings:**

We performed an integrative genomic analysis, incorporating whole exome sequencing (WES), determination of DNA copy number and DNA methylation, and transcriptome sequencing for 101 LUAD samples from the Environment And Genetics in Lung cancer Etiology (EAGLE) study. We detected driver genes by testing whether the nonsynonymous mutation rate was significantly higher than the background mutation rate and replicated our findings in public datasets with 724 samples. We performed subclonality analysis for mutations based on mutant allele data and copy number alteration data. We also tested the association between mutation signatures and clinical outcomes, including distant metastasis, survival, and tumor grade. We identified and replicated two novel candidate driver genes, POU class 4 homeobox 2 (*POU4F2*) (mutated in 9 [8.9%] samples) and *ZKSCAN1* (mutated in 6 [5.9%] samples), and characterized their major deleterious mutations. *ZKSCAN1* was part of a mutually exclusive gene set that included the RTK/RAS/RAF pathway genes *BRAF*, *EGFR*, *KRAS*, *MET*, and *NF1*, indicating an important driver role for this gene. Moreover, we observed strong associations between methylation in specific genomic regions and somatic mutation patterns. In the tumor evolution analysis, four driver genes had a significantly lower fraction of subclonal mutations (FSM), including *TP53 (p* = 0.007), *KEAP1* (*p* = 0.012), *STK11* (*p* = 0.0076), and *EGFR* (*p* = 0.0078), suggesting a tumor initiation role for these genes. Subclonal mutations were significantly enriched in APOBEC-related signatures (*p* < 2.5×10^−50^). The total number of somatic mutations (*p* = 0.0039) and the fraction of transitions (*p* = 5.5×10^−4^) were associated with increased risk of distant metastasis. Our study’s limitations include a small number of LUAD patients for subgroup analyses and a single-sample design for investigation of subclonality.

**Conclusions:**

These data provide a genomic characterization of LUAD pathogenesis and progression. The distinct clonal and subclonal mutation signatures suggest possible diverse carcinogenesis pathways for endogenous and exogenous exposures, and may serve as a foundation for more effective treatments for this lethal disease. LUAD’s high heterogeneity emphasizes the need to further study this tumor type and to associate genomic findings with clinical outcomes.

## Abstract 2

Maria Teresa Landi and colleagues report genomic tumor data for a cohort of patients with lung adenocarcinoma, focusing on implications for tumor initiation and distant metastasis.

## Author Summary

**Why Was This Study Done?:**

- Lung adenocarcinoma (LUAD) is the most common histologic subtype of lung cancer and causes more than half a million deaths worldwide annually.
- Genomic studies of LUAD can shed light on tumor initiation and progression and identify potential targets for treatment.

**What Did the Researchers Do and Find?:**

- We performed an integrative genomic analysis, incorporating whole exome sequencing (WES), DNA copy number and DNA methylation determination, and transcriptome sequencing in 101 LUAD samples. We replicated major findings using public genomic resources and combined all existing genomic data for an overall analysis of 825 LUAD samples.
- We identified two novel driver genes and characterized the driver events and types of mutations that have a stronger role in tumor initiation versus tumor progression.
- We found strong associations between DNA methylation and somatic mutation patterns.
- The total number of somatic mutations and the fraction of C→T transitions were associated with increased risk of distant metastasis.

**What Do These Findings Mean?:**

- We characterized LUAD genomic architecture and linked major genomic features with clinical outcomes.
- Tobacco smoking-related mutations appear to have a stronger role in tumor initiation, while mutations associated with endogenous processes are more prominent at a later stage of tumor development and are associated with tumor progression.
- Our findings highlight the complexity and heterogeneity of LUAD. In addition to new driver genes, we found some tumors with no exonic mutations in known lung cancer driver genes. This suggests that there are further drivers (genetic or epigenetic) to be identified, and larger numbers of samples need to be studied to fully capture LUAD genomic characteristics.

## Introduction

Lung cancer is the major cause of cancer mortality, causing approximately 1.38 million deaths worldwide annually [1]. Lung adenocarcinoma (LUAD) is the most common histologic subtype and accounts for more than 40% of lung cancer incidence. The absolute risk of distant metastasis in LUAD exceeds that of local recurrence at every disease stage, highlighting the systemic threat of the disease [2].

Multiple studies have been performed to characterize the genomes and transcriptomes of LUAD tumors. Array-based copy number aberration (CNA) studies [3,4] have identified important amplified genes, including *NKX2-1*, *TERT*, *EGFR*, and *MET*, and deleted genes, including *CDKN2A*. Targeted sequencing of protein-coding genes [5,6] and whole-exome sequencing (WES) [4,7–9] of tumor/normal or tumor/blood sample pairs have identified more than 20 mutated genes that showed positive selection in LUAD. Moreover, transcriptome sequencing has discovered recurrent gene fusions, including *EML4-ALK* [10], *KIF5B-RET* [11,12], and *ROS1* fusions [13]. These studies nominated driver genes and provided targets for treatment. For patients with *EGFR* mutations or *ALK* fusions, targeted kinase inhibitors have superior efficacy compared with the traditional chemotherapy [14].

Although many genes have been identified as potential drivers and targets for therapy, a large fraction of LUAD patients do not carry mutations in the set of most frequently mutated genes, suggesting substantial heterogeneity in the genomic drivers of LUAD. This observation highlights the need to sequence further LUAD samples to identify additional driver genes and targets for treatment.

In the current study, we sequenced the exome of tumor/blood sample pairs of 101 LUAD patients and the transcriptome of 80 LUAD tumor tissue samples from the Environment And Genetics in Lung cancer Etiology (EAGLE) study [15], in which a cohort of patients with LUAD were followed up for several years and comprehensive data on demographic, behavioral, and clinical characteristics were collected. Most patients were heavy smokers and were diagnosed at an early disease stage.

The study has several aims: to identify novel driver genes and gene fusions, to investigate the relationship between DNA methylation and somatic mutation signatures, to perform subclonality analysis, and to investigate the association of genomic features with exposures (e.g., tobacco smoking) and clinical characteristics (including survival, distant metastasis, tumor grade, and chronic obstructive pulmonary disease [COPD]).

## Methods and Materials

### Samples

The study protocol was approved by the institutional review board of the United States National Cancer Institute and the involved institutions in Italy. Informed consent was obtained for all subjects prior to study participation. We assayed 101 fresh-frozen stage I to IIIA lung adenocarcinoma tumor tissue samples from the EAGLE study [15], a large population-based, case–control study conducted in the Lombardy region of Italy between April 2002 and February 2005. All subjects were of Italian nationality between the ages of 35 and 79 years, official residents of the catchment area, and with no severe disease that could impede participation. Cases were newly diagnosed primary cancers, verified by tissue pathology. Lung tissue samples were snap-frozen in liquid nitrogen within 20 min of surgical resection. Surgeons and pathologists were present together in the surgery room at the time of resection and sample collection to ensure correct sampling of tissue from the tumor, adjacent lung tissue, and distant noninvolved lung tissue. The precise site of tissue sampling was indicated on a lung drawing and classified by the pathologists. For the current study, we selected 101 samples based on the histological characteristics (pure adenocarcinoma, not mixed types or undifferentiated cases), the presence of at least 50% tumor nuclei and less than 20% necrosis on histological review of the samples, and whether there was sufficient amount and quality of DNA and RNA needed for all the analyses.

All subjects in the EAGLE study were followed up in the same rigorous way. Detailed information on tumor characteristics, recurrence, treatment, and follow-up data were recovered from patients’ medical records at the end of the follow-up. After study completion, we identified follow-up visits and hospital admissions by linkage with the regionwide Regional Health Authority database of hospital admissions. Recurrence history was ascertained through December 31, 2010. Detailed clinical information was retrieved for each hospitalization and/or outpatient visit and reviewed by the clinical team that coded local recurrence and metastases [2]. The definition of local or distant recurrences followed the lung cancer staging as defined by the American Joint Committee on Cancer (AJCC), 7th Edition.

### Whole Exome Sequencing and Mutation Calling

Exome capture was performed using Agilent SureSelect Human All Exon V4, and whole exomes were sequenced on Illumina HiSeq 2000, both from Oxford Gene Technology (OGT). For paired-end reads from tumor and matched germline samples, we performed quality-based trimming and filtering by Trimmomatic (version 0.30). In this step, we removed the adapter and other Illumina-specific sequences from the reads. We removed the leading and trailing bases in a read if the quality scores were below 12. We also scanned from the 5ʹ end of the read with a 4-base wide sliding window and removed the 3ʹ end of the read when the average quality per base dropped below 15. We removed the reads below 36 bases after trimming. The sequence data were then aligned to the hg19 version of the human reference using Novoalign (version 3.00.05) and deduplicated by Picard (version 1.83). Local realignment around suspected sites of indels were performed using Genome Analysis Toolkit (GATK) IndelRealigner (version 2.8.1). These mapped sequence reads were used for mutation calling. Somatic single nucleotide variants (SNV) were identified using MuTect [16] (version 1.1.4) with default parameters. Indels were identified using GATK Indelocator. We identified APOBEC-mediated mutations as cytosine-to-thymine and cytosine-to-guanine substitutions in the TCW motifs (with W being either A or T) [17].

### Functional Annotation of Mutations Identified in Novel Driver Genes

Sequence variations were mapped to the corresponding genomic coordinates and inspected using the genome browsers of Ensembl (www.ensembl.org) and NCBI (www.ncbi.nlm.nih.gov). Protein sequence based information was extracted from the UniProt database (www.uniprot.org). Prosite domain based analysis was carried out using the Prosite database (http://prosite.expasy.org/). The 3-D structural coordinates for the relevant proteins were downloaded from the RCSB-PDB database (www.rcsb.org). For the 3-D fold–based analysis, protein models were built using the Phyre2 (http://www.sbg.bio.ic.ac.uk/phyre2/html/page.cgi?id=index) and I-TASSER (http://zhanglab.ccmb.med.umich.edu/I-TASSER) servers. Impact analysis predictions were carried out using MutationTaster (http://www.mutationtaster.org/), SIFT (http://sift.bii.a-star.edu.sg/), Provean (http://provean.jcvi.org/index.php), AVIA: Annotation, Visualization, and Impact Analysis (https://avia-abcc.ncifcrf.gov/apps/site/index; ver 2.0), and the Variant Effect Predictor (VEP) from Ensembl. The splice site variants were analyzed using Human Splicing Finder (vers 2.4.1 and 3.0). The Human Genome Variation Society (www.hgvs.org) was consulted for reporting variations.

### Transcriptome Sequencing and Gene Fusion Analysis

Transcriptome sequencing of 80 tumor samples with RIN (RNA Integrity Number) ≥ 6 was performed on the Illumina HiSeq2000/2500 platform with 100 bp paired-end reads. The remaining 21 tumor samples had RNA with poor quality or amount and were not used for this analysis. Sequence reads were mapped by the Mapsplice algorithm with default parameters. We used the generic annotation file (version TCGA.hg19.June2011.gaf) to annotate genes and exons. Candidate gene fusion events were nominated by DeFuse [18]. The fusions were excluded if either break point was located in intron regions or within 100 kb of the same chromosome. After filtering, we selected 11 fusion events for validation. RNA extracted from patient samples was reverse transcribed with SuperScript III (Life technologies). Each cDNA library was subsequently amplified using fusion specific primers. Amplicons were electrophoresed and products of sizes that were similar to what was predicted were purified using Ampure XP (Epicentre). Purified amplicons were end-repaired, barcoded, adapted and pooled, and subsequently sequenced on an Ion Personal Genome Machine (PGM) sequencer (Thermo Fisher Scientific). Sequence data were uploaded to the Seven Bridges Genomics platform. FastQ sequence data were converted to BAM file formats and fusion transcripts were detected using STAR and Chimera pipelines. Results were filtered and analyzed using the Picard Alignment summary pipeline.

### DNA Methylation Profiling and Analysis

Bisulphite treatment and Illumina Infinium HumanMethylation450 BeadChip assays [19] were performed to profile the fresh-frozen tumor samples in the EAGLE cohort. Raw methylated and unmethylated intensities were background-corrected and dye-bias-equalized to correct for technical variation in signal between arrays. For background correction, we applied a normal-exponential convolution, using the intensity of the Infinium I probes in the channel opposite their design to measure nonspecific signals. Dye-bias equalization used a global scaling factor computed from the ratio of the average red and green fluorescing normalization control probes. For each CpG probe in the platform, the DNA methylation level was summarized as the fraction of signal intensity obtained from the methylated beads over the total signal intensity. After excluding CpG probes annotated with genetic variants (single nucleotide polymorphisms or copy number variations), in repetitive genomic regions or on the X chromosome, 338,730 CpG probes remained for analysis. Each CpG probe was annotated as in CpG island (denoted as CGI), nonCGI (including shores and shelves) or “open-sea”. Each CpG probe was also annotated as in promoter (TSS200, TSS1500, and first exon), body, 3ʹUTR in a specific gene, or annotated as intergenic. A CpG probe may have multiple different annotations depending on the transcripts used for annotation.

### Copy Number Alteration Analysis

Detecting CNAs is crucial for characterizing tumor genomes and also for estimating tumor purity and subclonality. We profiled CNAs for tumor samples using Illumina HumanOmniExpress SNP arrays. The majority of lung tumor genomes were disrupted by many CNAs with complex clonality (see example in S1A Fig), which made the segmentation difficult based on the intensity data. The B allele frequency (BAF) data for the heterozygous SNP probes provided a cleaner segmentation for CNAs (S1A Fig). Thus, we used Nexus (http://www.biodiscovery.com/nexus-copy-number/) to segment the cancer genome based on BAF data, followed by manual verification. For each segment, the sequencing read ratio between the tumor DNA sample and the matched germline DNA sample was used to determine whether it was amplification, hemizygous deletion (CN1), homozygous deletion (CN0), loss of heterozygosity (LOH), or copy neutral (CN2). We identified genomic regions significantly disrupted by CNAs using GISTIC [20,21]. We estimated tumor purity and subclonality based on detected deletions and LOH events using a Gaussian mixture model. Amplifications were excluded from the analysis because of their large amplitude variability. Details are presented in S1 Text and S1 Fig. We note that the estimated purity may be different from the histology-based count of tumor nuclei, which was manually determined and subject to human error.

### Subclonality Analysis for Somatic Point Mutations

A clonal SNV is a point mutation carried by all tumor cells, while a subclonal mutation is carried by a fraction of tumor cells. Previously developed methods (e.g., [22]) determine subclonality of a SNV by modeling the mutation allele fraction (MAF) and the copy number of the locus. However, these methods assume that CNAs at the locus are clonal to simplify the problem. This assumption is expected to cause incorrect statistical inference. Here, we extend the reported method [22] to infer whether a SNV is clonal or subclonal by accounting for tumor purity, CNA, and its subclonality (S2 Fig and S2 Text). Because of the difficulty in estimating the absolute copy number and the subclonality for amplifications, we restricted our analysis to mutations in normal, deleted, and LOH regions. For each gene, or a specified set of somatic mutations, we calculated the fraction of subclonal mutations out of all somatic mutations across patients.

### Statistical Analyses

This study performed integrative genomic analysis based on a set of lung cancer patients and had no prospective clinical protocol or analysis plan. The associations between genomic features (total number of somatic mutations, fractions of nine point mutation types, mutation status of *KRAS* and *TP53*, number of fusion events) and clinical outcomes (survival, distant metastasis, and local relapse) were assessed by the Cox regression model, with significance evaluated by the log-rank test. The association for COPD was evaluated by logistic regression. The association for tumor grade was evaluated using ordinal regression. All analyses were adjusted for age, sex, stage, and, as specified, also by smoking behaviors (smoking status, cigarettes per day, and smoking duration) in some analyses. We identified statistically significant associations by controlling the false discovery rate (FDR) <5%. The associations between mutation signatures and covariates (e.g., smoking behaviors) were assessed by the Wilcoxon rank sum test or Fisher’s exact test. All remaining statistical analyses were performed using the R package (http://www.r-project.org). *p*-values reported in the manuscript are nominal *p*-values.

We evaluated the significance for selective advantage of somatic mutations for each gene using MutSigCV [23] and nominated significant genes by controlling FDR <5%. MutSigCV is a program for testing whether the mutation rate of nonsynonymous mutations of a target gene is significantly higher compared with the background silent mutation rate by combining mutation data across patients. MuSigCV appropriately accounts for the heterogeneity of mutation rate across patients and mutation types. To increase the statistical power, MutSigCV tries to identify a “bagel gene set” to better estimate the background mutation rate. Here, a “bagel gene set” for a target gene refers to the genes with similar background mutation rate by matching gene expression levels and DNA replication time. The identified novel driver genes were replicated using MutSigCV in the 724 samples pooling the LUAD datasets in TCGA [4] and the Broad Institute study [7]. The mutual exclusivity for candidate driver genes was assessed using MEGSA [24] and mutex [25]. MEGSA aims to identify a mutually exclusive gene set (MEGS) by a likelihood ratio statistic for assessing mutual exclusivity, a model selection procedure to identify the optimal MEGS, and a permutation procedure for evaluating global significance. Mutex is a program for identifying MEGS by restricting gene sets with common downstream targets.

## Results

### Patient Characteristics

Table 1 summarizes the study subjects’ characteristics. Eight patients had neoadjuvant chemotherapy before surgery. The study included 7 never smokers, 42 former smokers, and 51 current smokers. Out of the 93 ever smokers, most were heavy smokers (averaging 21.5 cigarettes per day) for a long time (42.7 years on average). Thirty-eight patients were alive at the time of last follow-up. As expected, stage was significantly associated with survival (*p* = 0.0007 for stage II and *p* = 0.005 for stage IIIA, with stage I as baseline). Forty patients developed distant metastases and 17 patients developed local relapse.

**Table 1:** Distribution of demographic and clinical variables of 101 lung adenocarcinoma patients.

Age at first diagnosis (mean, range)	65.3 (44–79)
Sex	
Male	83
Female	18
Smoking status	
Never	7
Former	42
Current	51
Missing	1
Cigarettes per day	
≤10	15
>10, ≤20	48
>20, ≤30	16
>30	12
Missing or never smokers	10
Mean (standard deviation)	21.5 (9.5)
Cigarette smoking duration	
≤30 years	9
>30, ≤40	35
>40, ≤50	25
>50 years	22
Missing or never smokers	10
Mean (s.d.)	42.7 (10.7)
Tumor stage	
IA	26
IB	25
IIA	21
IIB	9
IIIA	20
Chemotherapy	
yes	8
no	93
Distant metastasis	40
Local recurrence	17

### Somatic Mutations Detected in WES and Their Associations with Smoking Characteristics

**Fig 1:**
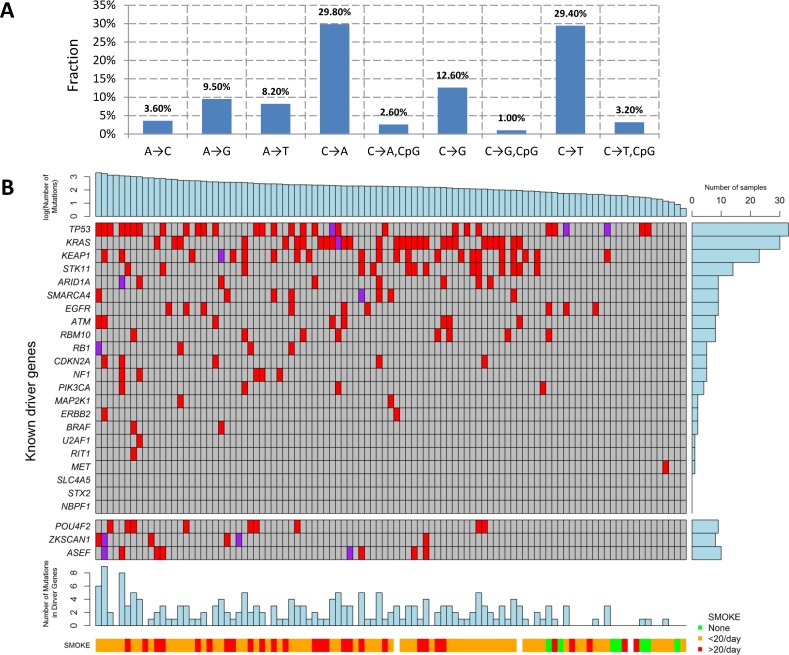
Somatic mutations of lung adenocarcinoma in EAGLE data. **(A)** Distribution of point somatic mutations across nine mutation types. **(B)** The top panel shows the number of nonsilent mutations detected by whole-exome analysis for 101 EAGLE samples. Tumor samples were arranged from left to right by the number of nonsilent mutations. The middle panel shows the mutations for previously reported significantly mutated genes based on the TCGA data, reported in the TumorPortal website. The next panel shows the mutations for the three new driver genes. The bottom panels show smoking status. The right panel shows the frequency of nonsilent mutations in EAGLE data for each driver gene. Each column represents one patient.

We performed WES for 101 LUAD tumor/blood sample pairs. The average WES coverage was 107 for tumor and 58 for germline DNA samples. After excluding low quality calls, we identified 459 indels (16 at a splice site) and 40,704 exonic point mutations, including 28,200 missense, 2,129 nonsense, 87 frameshift, 1,264 splice-site, and 10,140 silent mutations. The average somatic mutation rate was 11.3 per megabase (Mb) in exonic regions, with 7.9 nonsynonymous and 2.8 synonymous mutations on average, consistent with previously reported rates [4,7]. The mutations are listed in S1 Table. The distribution of the point mutations is reported in Fig 1A.

Smoking status (ever versus never), duration, and intensity were significantly associated with increased total number of somatic mutations (TNSM) (*p* = 0.007, 0.02, and 0.01, respectively, Wilcoxon rank sum test). Neither disease stage nor chemotherapy history was significantly associated with TNSM.

The mutation types were dominated by C→A and C→T signatures (Fig 1A). We investigated whether the fraction of each mutation type was associated with smoking behaviors (S2 Table). Compared with never smokers, smokers had a significantly increased fraction of C→A transversions (*p*_CpG_ = 0.0007 and *p*_nonCpG_ = 0.0014) and a significantly decreased fraction of C→T transitions (*p*_CpG_ = 0.0001 and *p*_nonCpG_ = 0.0005). Here, *p*_CpG_ and *p*_nonCpG_ denote the association *p*-values for mutations inside and outside of the CpG context, respectively. Number of cigarettes per day was also significantly associated with an increased fraction of C→A transversions (*p*_CpG_ = 0.006 and *p*_nonCpG_ = 0.004) and reduced fraction of C→T transitions (*p*_CpG_ = 0.006 and *p*_nonCpG_ = 0.004). Smoking duration was significantly associated with a reduced fraction of C→T transitions within the CpG context (*p* = 0.004). The fraction of nucleotide transversions was associated with smoking status (ever versus never, *p* = 1.7×10^−4^, average fraction = 0.31 for never and 0.62 for ever smokers), consistent with previous reports [4,7]. We did not detect significant differences between former and current smokers.

The fractions of APOBEC-mediated mutations ranged from 0% to 40.7% across 101 samples (mean 10.5%), similar to the TCGA (mean = 11.1%) [17] and Broad Institute (mean = 9.5%) [7] datasets. APOBEC-mediated mutations were depleted in nonsynonymous mutations (*p* = 1.5×10^−9^, Fisher’s exact test): 12.6% of synonymous mutations and 10.4% of nonsynonymous mutations were APOBEC-related. The fraction of APOBEC mutations was not significantly associated with sex, age, or disease stage, nor with smoking status, intensity, or duration.

### Significantly Mutated Genes in Lung Adenocarcinoma

The TumorPortal [26] reports 37 significantly mutated genes for LUAD based on 398 TCGA samples. A pooled analysis of 412 LUAD samples (including 231 TCGA samples and 181 Broad samples) reported 18 significantly mutated genes [4]. Among these genes, 10 were mutated in the EAGLE sample with frequency greater than 5% (Fig 1B): *TP53* (33.7%), *KRAS* (30.7%), *KEAP1* (23.8%), *STK11* (14.9%), *ARID1A* (7.9%), *RBM10* (8.9%), *SMARCA4* (8.9%), *EGFR* (8.9%), *ATM* (7.9%), and *RBM10* (7.9%). For over 20% of the EAGLE samples, the previously identified LUAD genes had no nonsynonymous mutations.

We identified three novel significantly mutated genes: *POU4F2* (*p* = 2.1×10^−6^), *ZKSCAN1* (*p* = 4.1×10^−6^), and *ASEF* (*p* = 3.1×10^−5^) (Fig 1B) by controlling FDR <0.05. After excluding samples from the 8 patients who had received chemotherapy, the three genes remained statistically significant (*p* = 5.2×10^−6^ for *POU4F2*, *p =* 1.9×10^−5^ for *ZKSCAN1*, and *p* = 3.2×10^−4^ for *ASEF*). All mutations in the three genes were validated by Sanger sequencing. We extracted the mutation data in these three genes from 542 TCGA LUAD samples and 182 Broad LUAD samples with WES data. S3 Table summarizes the number of synonymous and nonsynonymous mutations in these datasets. We tested the significance of the three genes using MutSigCV in the 724 samples pooling the two WES datasets. *POU4F2* (*p* = 1.3×10^−4^) and *ZKSCAN1* (*p* = 0.029) were significantly mutated, while *ASEF* (*p* = 0.46) was not. Detailed investigation suggested that the lack of significance for *ASEF* was due to the high synonymous mutation rate in the “bagel” gene set [23], which was used for estimating the background silent mutation rate. In addition, *POU4F2* and *ZKSCAN1* were not detected as significant driver genes in previous studies because they had a lower rate of nonsynonymous mutations.

**Fig 2:**
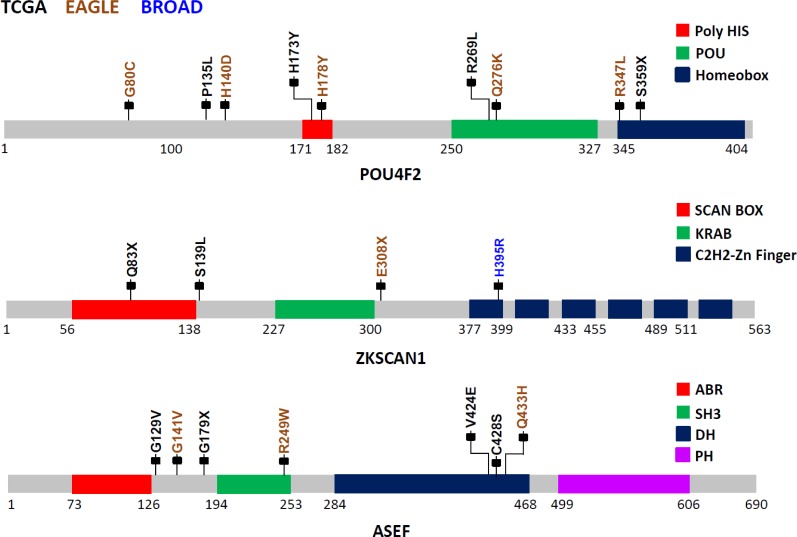
Somatic mutations in three LUAD candidate driver genes (*POU4F2*, *ZKSCAN1*, and *ASEF*) in EAGLE, TCGA and Broad Institute studies. The protein sequences from these three genes are schematically described using grey bars along with their respective structural and functional domains in color-coded blocks. Each mallet represents an independent nonsilent mutation with potential functional relevance in the three studies (the complete list of mutations is reported in S1 Table). Numbers below each sequence representation mark the total length of the transcript, the domain ranges, and the locations of mutations.

*POU4F2*, also known as *BRN-3B*, is a member of the POU-domain transcription factor family. Although 3-D structural data are not available for this protein, domains have been predicted based on amino acid sequences, beginning with amino acid 250 (aa250; POU:250–327; HOMEOBOX:345–404). Because of the homology between the two C-terminal conserved domain segments, POU4F2 is considered to be part of the Pit-Oct-Unc family. Therefore, the human Oct-1 protein, with an experimentally determined 3-D conformation, can be used as the basis for POU4F2 structure-based analysis. Based on the Oct1 structure, several mutations identified in this study are noteworthy (Fig 2): S359X (TCGA), which could lead to a nonsense-mediated decay (NMD) or a truncated protein with the loss of most of the Homeobox domain, and R269L (TCGA), Q276K (EAGLE), and R347L (EAGLE) variations, which could modify the potential DNA binding residues, eventually disrupting protein function. Using sequence-based analysis, other mutations could also be important, such as G80C (EAGLE), which is part of the glycine/serine motif, where the newly introduced residue cysteine has the capability to form intra- or inter-S-S bonds that could modify protein structure and/or function. Moreover, histidine residue modifications such as H173Y (TCGA) and H178Y (EAGLE) belong to the homopolymeric histidine tract (Q12837:171-HHHHHHHHHHH-182) that is often associated with essential DNA- and RNA-related functions [27], as well as the P135L (TCGA) and A140D (EAGLE) variants, which are not commonly observed substitutions based on the BLOSUM62 score and are also closely located to the previously identified histidine repeat region. Finally, the intronic variant ENST00000281321.3:c.288+1 G→A, being located in the exon–intron border, could potentially affect normal splicing (Fig 2).

The zinc finger with Kruppel-associated box (KRAB) and SCAN (or leucine-rich region [LeR]) domains 1 (*ZKSCAN1* or *ZNF139*) gene encodes a transcriptional regulator protein and is a member of the KRAB subfamily of zinc finger proteins. Although no experimental 3-D structural information for ZKSCAN1 is available, sequence-based analysis suggests that several mutations identified in this study can disrupt ZKNSCAN1 function: e.g., Q83X (TCGA) and E308X (EAGLE) are potential NMD mutations, and, if expressed, they could result in a truncated protein with the loss of all six zinc finger domains (C2H2-1 to C2H2-6); variation chr7:99621041G>T (TCGA) occurs as the last nucleotide in the intron 1–2, can be considered as a splice acceptor variant, and most likely has an impact on alternative splicing; S139L (TCGA), which adds a leucine to the end of a leucine-rich domain (prosite: PS50804); and H395R (Broad Institute) modification, which occurs in the first zinc finger domain, possibly disrupting the Zn^2+^ ion binding site (Fig 2).

The APC-stimulated guanine nucleotide exchange factor (*ASEF*) gene, also known as Rho guanine nucleotide exchange factor (GEF) 4 (*ARHGEF4*), is a regulator of the cytoskeleton and cell migration [28]. Detailed description of the mutations and possible biological implications are in S3 Text.

### DNA Methylation and Somatic Mutation Signature

In an integrated analysis of 93 samples with both DNA methylation and WES data, we performed genome-wide analysis to identify CpG probes with methylation levels associated with somatic mutation patterns, including TNSM, fraction of transversions (FT), fraction of APOBEC-related mutations (FA), and fractions of the nine point mutation types. Associations were adjusted for smoking status, age, disease stage, and sex. We identified 100 CpG probes significantly associated with TNSM (*p* < 1.4×10^−7^, Bonferroni correction) in the EAGLE dataset. All findings were replicated in the 393 TCGA samples with both WES and methylation profiles at *p* < 0.05 and with the same direction of association.

**Fig 3:**
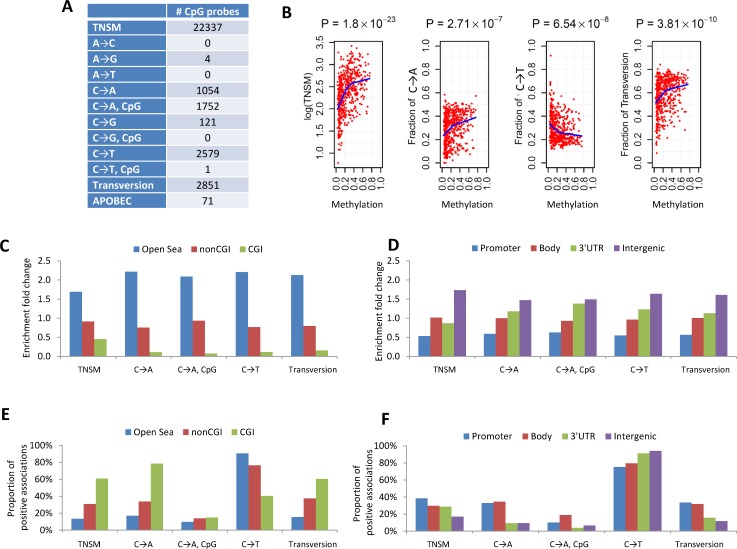
The associations between DNA methylation and somatic mutation signatures based on EAGLE and TCGA data. **(A)** The number of CpG probes significantly associated with the TNSM and the fractions of various types of point mutations (*p* < 1.5×10^−7^, based on Bonferroni correction). **(B)** CpG probe cg00042837 was strongly associated with TNSM, the fractions of C→A mutations, C→T mutations, and transversions. Each point represents one sample. The blue line was generated by “lowess,” a nonparametric statistical procedure for nonlinear regression. **(C)** The enrichment fold change of CpG probes mapping to different categories in the association with somatic point mutation types. “CGI” represents CpG island regions; “NonCGI” includes shore and shelf regions. **(D)** The enrichment fold change of CpG probes mapping to different gene regions in the association with point somatic mutation types. **(E)** and **(F)** show The proportion of identified CpG probes showing positive associations with different somatic point mutation types.

To increase the statistical power, we performed a meta-analysis by combining the EAGLE and TCGA samples with both WES and methylation data (*n* = 486 samples in total). This analysis identified over 1,000 CpG probes significantly associated with TNSM, FT, the fraction of C→A mutations (both within and outside CpG regions), and the fraction of C→T mutations outside CpG regions (Fig 3A) (*p* < 1.5×10^−7^, Bonferroni correction). Many specific CpG probes were associated with multiple mutation traits (CpG probe cg00042837 is presented as an example in Fig 3B).

We investigated whether CpG probes in each annotation category were enriched for associations with each somatic point mutation trait. As an example, out of the 110,542 isolated CpG probes (referred to as “open-sea” [29]), 12,339 (11.2%) were significantly associated with TNSM. On average, 22,337 (6.6%) of all 338,739 CpG probes were significantly associated with TNSM. Thus, the enrichment fold change was calculated as 11.2%/6.6% = 1.7 for CpG probes mapping to open-sea regions. Fig 3C and 3D report the enrichment fold change. The CpG probes mapping to open-sea and intergenic regions were strongly enriched for the associations with almost all investigated point mutation types; the CpG probes mapping to CpG island and gene promoter regions were strongly depleted for associations.

We calculated the fraction of positive associations of CpGs over each somatic point mutation type (Fig 3E and 3F). The fractions of positive associations varied substantially across different mutation types and different CpG categories. For TNSM, 13.4% of associated CpG probes mapping to open-sea regions showed positive associations, while the fraction was 60.8% for CpG probes mapping to island regions.

### Landscape of CNAs, Tumor Purity, and Clonal and Subclonal CNAs

We identified 1,931 deletions, 1,345 amplifications, and 471 LOH events with lengths greater than 1,000,000 base pairs. On average, each sample had 19.1 deletions, 13.3 amplifications, and 4.7 LOH events. Approximately 40% of the genome was disrupted by CNAs on average. The fractions of genomes disrupted by CNA were not significantly associated with smoking behaviors, disease stage, sex, or chemotherapy.

GISTIC analysis [20] restricted to large-scale deletions (>50% of chromosome arms) identified the majority of previously reported regions at FDR <0.05 [3] and new regions, including 16p and 16q. The analysis also replicated most of previously reported amplifications, including the 1q region for large-scale amplification events and the 1q21.2, 5p15.33, 8q24.21, 12p12.1, 12q15, 14q13.3, and 19q.12 regions for focal amplification (S4 Table and S3 Fig).

Tumor purity was on average 48.5% (standard deviation = 18.9%). We estimated that 56.6% of deletions and 90.3% of LOHs were subclonal. For each sample, we calculated the fraction of subclonal deletions and/or LOHs and examined the association with covariates. The fractions were nominally associated with smoking status (never versus ever, *p* = 0.02, Wilcoxon rank sum test) but not with other variables such as sex, disease stage, chemotherapy, distant metastasis, local recurrence, or survival.

### Clonal and Subclonal Somatic Mutations

**Fig 4:**
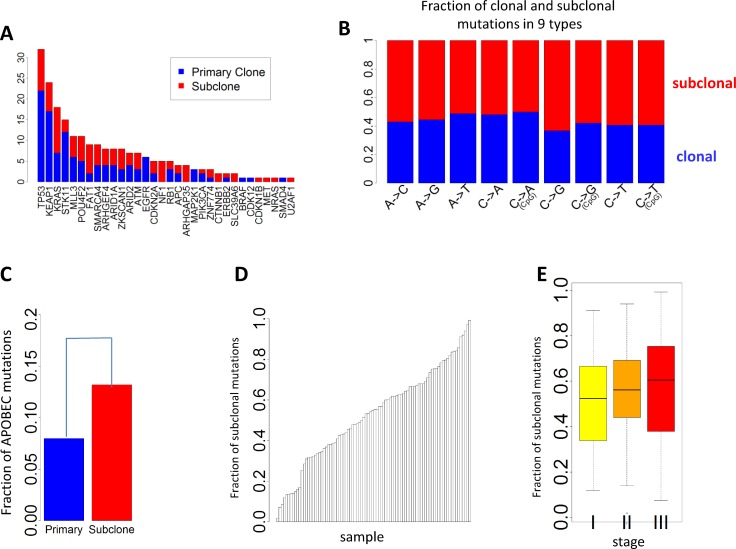
Clonal and subclonal point mutations in EAGLE data. Mutations in amplification regions were not included in the analysis. **(A)** The number of clonal and subclonal mutations in 37 driver genes for lung adenocarcinoma. **(B)** Fraction of clonal and subclonal mutations in each of the nine point mutation types. **(C)** The fraction of APOBEC-mediated mutations significantly differed in clonal and subclonal mutations. **(D)** Estimated fraction of subclonal mutations for each sample. **(E)** Estimated fractions of subclonal mutations for patients at different tumor stages.

Subclonal mutations accounted for 55.5% of all mutations that were successfully classified. The FSM was significantly lower for reported driver genes (FSM = 46.8%) than nondriver genes (FSM = 55.5%) (*p* = 0.011, Fisher’s exact test), consistent with a recent Pan-Cancer analysis [30]. Fig 4A presents the numbers of clonal and subclonal mutations for 32 genes in LUAD with mutations in our data (other driver genes with no mutations in EAGLE samples were not included). Four driver genes had significantly lower FSM compared with the overall FSM distribution based on Fisher’s exact test: *TP53* (*p* = 0.007), *KEAP1* (*p* = 0.012), *STK11* (*p* = 0.0076), and *EGFR* (*p* = 0.0078). FSM significantly differed between the nine types of point mutations (Fig 4B). APOBEC-mediated mutations were significantly enriched in subclonal mutations (Fig 4C) (*p* < 2.5×10^−50^, Fisher’s exact test): 13.2% of subclonal mutations and 8.0% of clonal mutations were APOBEC mutations. FSM did not differ significantly between synonymous and nonsynonymous mutations (*p* = 0.98, Fisher’s exact test). FSM differed significantly between C→A mutations and C→T mutations (*p* = 2.85×10^−27^, Fisher’s exact test).

Fig 4D reports the FSM for all patients. FSM did not show significant association with smoking behaviors (*p* = 0.59 for smoking status, *p* = 0.56 for duration, and *p* = 0.97 for cigarettes per day), neoadjuvant chemotherapy (*p* = 0.78), or disease stage (*p* = 0.28). Although not significant, more advanced patients showed a higher FSM (average FSM: 49.6% for stage I, 55.9% for stage II, and 56.7% for stage III) (Fig 4E).

### Mutual Exclusivity Analysis

**Fig 5:**
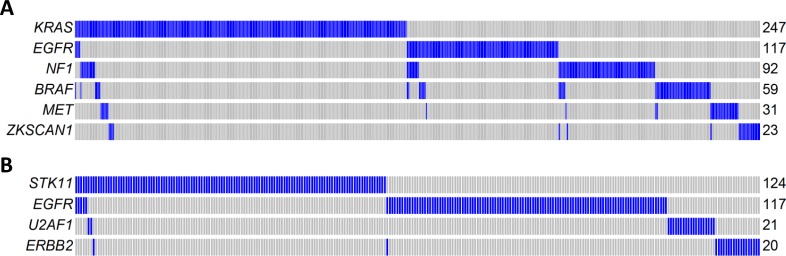
Mutual exclusivity of driver genes detected in 825 patients combining TCGA, Broad Institute, and EAGLE WES of lung adenocarcinoma. **(A)** A MEGS with six genes covering 60.3% of patients. Samples without nonsynonymous mutations in these six genes are not shown. Samples labelled as blue carry a nonsynonymous mutation in the gene region, while samples labelled as gray do not carry a synonymous mutation in the gene region. **(B)** A MEGS with four genes covering 33.3% of patients. Samples without nonsynonymous mutations in these four genes are not shown.

Mutual exclusivity analysis investigates the relationship between driver genes and identifies pathways or gene sets that may exert similar oncogenic functions. To increase the statistical power, we pooled WES data from three LUAD studies: TCGA, Broad Institute, and EAGLE, totaling 825 subjects. The analysis was performed for nonsynonymous mutations of 32 significantly mutated genes with mutation frequencies greater than 2% using MEGSA [24]. All reported *p*-values were adjusted for multiple comparison using 10,000 permutations. We identified four significant but overlapping MEGS, with *EGFR* included in three. The largest and most significant MEGS (*p* < 10^−4^) had 6 genes (*BRAF*, *EGFR*, *KRAS*, *MET*, *NF1*, and *ZKSCAN1*), with mutations covering 60.3% of patients (Fig 5A). This largely overlapped with the previously reported MEGS (*BRAF*, *EGFR*, *KRAS*, *MET*, *NF1*, *ERBB2*, *ROS1/ALK/RET*, *MAP2K1/HRAS/NRAS*) that was discovered by using point mutations, fusions, and amplification events [4]. Importantly, *ZKSCAN1* is a novel candidate gene identified in our study. Another highly significant MEGS involving *EGFR* is (*EGFR*, *STK11*, *U2AF1*, *ERBB2*), with mutations in 33.3% of LUAD (Fig 5B). The other two MEGS were pairwise: (*KRAS*, *TP53*), with mutations covering 69.5% patients, and (*EGFR*, *KEAP*), with mutations covering 30.3% patients. We also performed analysis using mutex [25] to search for special MEGS with genes sharing a common downstream target. This analysis identified (*EGFR*, *KRAS*, *SMARCA4*) as a significant MEGS, with a common downstream target gene *PIK3CD*.

### Gene Fusions in Transcriptome Sequencing

We sequenced the transcriptome of 80 mRNA samples and one Japanese LUAD mRNA sample previously reported with a *KIF5B-RET* fusion [11] as a positive control. Using defuse [18], we successfully detected the *KIF5B-RET* fusion and identified 49 fusions with both breaking points located in exonic regions of two different chromosomes or with breaking points less than 100 kb on the same chromosome (S5 Table). Twenty-four subjects had at least 1 fusion. We selected 11 fusions with primers available for experimental validation and successfully validated 10 (S5 Table).

The number of fusions was significantly associated with sex (*p* = 0.023; females had 0.51 more fusions than males on average) but not significantly associated with age at diagnosis, smoking behaviors, or disease stage. Consistent with a previous report [31], the number of fusion events was suggestively associated with reduced survival (*p* = 0.069) after adjusting for disease stage, age, and sex. S4–S6 Figs reports three experimentally validated fusions as examples: *FOXK2-KRT20*, *FOXN1-BLMH*, and *RUNX1-FARS2*.

### Associations between Genomic Alterations and Clinical Outcomes

We analyzed the association between genomic alterations and clinical outcomes, including survival, spirometry-based COPD, tumor grade, distant metastasis [2], and local relapse after surgery in the EAGLE study. The examined genomic alterations included TNSM, fraction of transversions, fractions of nine types of point mutations, fractions of the genome covered by CNAs, and mutational status of *TP53* and *KRAS*, which were most frequently mutated in LUAD. The associations were evaluated in 93 samples without presurgical neoadjuvant chemotherapy and were adjusted for sex, age, and disease stage. We tested 70 (= 5 traits × 14 genomic features) hypotheses and identified statistically significant associations by controlling for an FDR <5%.

**Fig 6:**
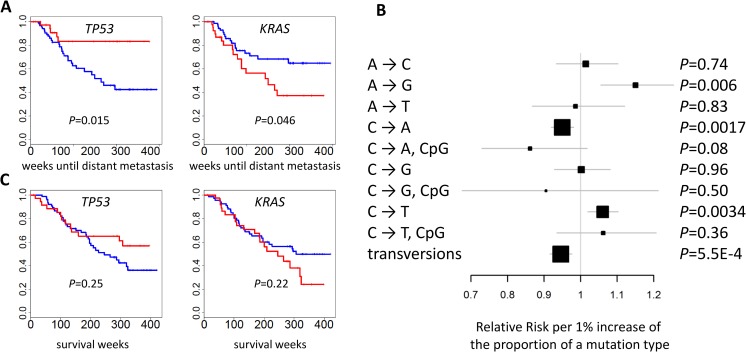
Association between genomic features and clinical outcomes. **(A)** The mutational status of TP53 and KRAS and the time of developing distant metastasis. *p*-values were two-sided. Red: mutated; blue: not mutated. **(B)** The association between the fraction of nine point mutation types and overall transversions and the time of developing distant metastasis after initial diagnosis. Relative risks and their 95% confidence intervals were estimated based on a Cox regression model adjusted for age, sex, and disease stage. *p*-values were two-sided. **(C)** Cancer-free survival was not associated with the mutational status of TP53 or KRAS. *p*-values were two-sided. Red: mutated; blue: not mutated.

The mutational status of *TP53* (*p* = 0.015) and *KRAS* (*p* = 0.046) was associated with the risk of developing distant metastasis (Fig 6A). Further adjustment for smoking status and intensity slightly improved significance: *p* = 0.014 for *TP53* and *p* = 0.015 for *KRAS*. The risk of developing distant metastasis was significantly associated with TNSM (*p* = 0.0039), the fraction of transversions (*p* = 0.00055), and the fractions of three mutation types (*p* = 0.006 for A→G; *p* = 0.0017 for C→A; *p* = 0.0034 for C→T) (Fig 6B). These associations remained significant after further adjusting for smoking status and intensity: *p* = 0.0039 for TNSM, *p* = 0.0016 for the fraction of transversions, *p* = 0.0017 for the fraction of A→G mutations, *p* = 0.0027 for the fraction of C→A mutations, and *p* = 0.010 for the fraction of C→T mutations. The fraction of APOBEC-mediated mutations was not significantly associated with risk of developing metastasis (*p* = 0.49). The mutation status of *POU4F2* or *ZKSCAN1* was not significantly associated with clinical outcomes.

While TNSM was significantly associated with an increased risk of developing distant metastasis, such an association was not significant (*p* = 0.33) if restricted to the number of mutations in the LUAD genes summarized in Fig 1B. Restricting to nonsynonymous mutations decreased the significance of these associations. This suggests that both synonymous and nonsynonymous mutations in unidentified “driver” genes could contribute to the risk of developing metastasis.

We reexamined the associations separately for clonal and subclonal mutations and did not detect additional significant associations. In fact, restricting to clonal or subclonal mutations reduced the associations for the risk of developing distant metastasis, suggesting that both clonal and subclonal mutations contributed to metastatic disease.

No genomic feature was significantly associated with survival. Previous studies reported that *TP53* or *U2AF1* mutation events were significantly associated with reduced survival [7,32]. We could not evaluate the association of *U2AF1* because only one patient in EAGLE carried a mutation in that gene. Our data did not support the association for *TP53* (*p* = 0.25) (Fig 6C), possibly because of limited statistical power due to a small sample size. A network smoothing method [33] was recently proposed to stratify tumors based on somatic mutations and identified clusters showing significantly different survival in the TCGA data. However, this analysis did not produce clusters with differential survival distribution in our data. No significant associations were detected for COPD, local relapse, or tumor grade.

## Discussion

### Sequencing Analysis Identified Novel Driver Genes

As expected, we found a high rate of somatic mutations in lung adenocarcinoma (LUAD). Moreover, C→A and the overall transversion ratio were associated with smoking phenotypes. We identified three novel driver genes on the basis of frequency and types of mutations in *POU4F2*, *ZKSCAN1*, and *ASEF*, and the apparently significant occurrence of *POU4F2* and *ZKSCAN1* mutations in LUAD was replicated in a pooled analysis of TCGA and Broad Institute WES studies. By sequencing additional LUAD samples from the same Italian population, we found no evidence that the mutation frequencies of the three genes differ between populations.

*POU4F2* is overexpressed in breast cancer and neuroblastoma cells, in which it promotes tumor growth [34,35]. In breast cancer, POU4F2 can repress *BRCA1* expression [36] and interact with estrogen receptor alpha to enhance its activity [35]. POU4F2 can also induce the expression of *CCD1* and *CDK4* in the context of proliferation and cell cycle progression [34,37], confer migratory and multidrug resistance properties to breast cancer in in vivo models [38], and cooperate with TP53 to increase transcription of pro-apoptotic genes [39]. Importantly, growth factors like the epidermal growth factor (EGF) can stimulate the activity of the *POU4F2* promoter and subsequently its mRNA and protein expression, which in turn can affect POU4F2 target genes influencing growth and behavior of cancer cells.

*ZKSCAN1* (or *ZNF139*) has been reported to have increased expression in gastric cancer cells [40–43]. In gastric cell lines, it has also been shown to promote migration, invasion, and multidrug resistance [41,42]. Knockdown of *ZKSCAN1* in GC cell lines leads to changes in expression of *MMP2*, *MMP9*, *ICAM1*, and *TIMP1*, impacting cell adhesion and matrix metalloprotease activities [42]. BCL-2 and other modulators of apoptosis are also among the ZKSCAN1 targets [41]. We identified *ZNSCAN1* among a set of genes that are mutually exclusively mutated in lung adenocarcinoma and are all involved in the RTK/RAS/RAF pathway, including *EGFR*, *BRAF*, *KRAS*, *MET*, and *NF1*. This suggests that *ZKSCAN1*, like the other genes in this pathway, has an important role in lung carcinogenesis.

### Strong Association between DNA Methylation and Somatic Mutation Signatures

We found a strong association between CpG methylation probes and somatic mutations, with over 1,000 CpG probes associated with C→A transversions within or outside CpG island regions and over 2,000 CPG probes associated with C→T mutations outside the CpG island regions. An elevated rate of somatic mutations has been observed in hypermethylated tumors in other cancer types [44]. In our study, the CpG probes mapping to open-sea and intergenic regions were strongly enriched for the associations with almost all investigated mutation types, but the largest positive association across all gene regions was with C→T mutations. In contrast, the small proportion of methylated CpG probes mapping to CpG islands were positively associated with both C→A and C→T mutations, suggesting a different function of methylation across point mutation types.

Multiple mechanisms could operate at methylated CpG sequences to produce mutational hotspots. The most well-known pathway involves spontaneous deamination of 5-methylcytosine to form thymine as T/G mismatches. If not repaired, mostly by thymine DNA glycosylase (TDG) or methyl-CpG binding domain protein (MBD4), these mismatches may induce C→T transition mutations by polymerase bypass [45]. Unmethylated cytosine can also undergo deamination with subsequent formation of uracil, which is typically rapidly removed by the ubiquitous uracil-DNA glycosylase enzymes but also by TDG and MBD4. TDG and MBD4 may thus counteract the mutagenic consequences of deamination of cytosine or 5-methylcytosine [46–48] and may also repair oxidized and adducted pyrimidines. We tested whether the expression of *TDG* or *MBD4* was associated with the frequency of C→T transitions in TCGA LUAD. We found that expression was negatively correlated with C→T mutations, particularly for TDG (*r* = –0.26, *p* = 10^−7^), supporting a role for inefficient DNA repair in the etiology of the observed C→T mutations. Another pathway, in the presence of 5-methlcytosine at CpG sequences, involves the formation of DNA adducts at the neighboring guanines. DNA adducts are typically associated with polycyclic aromatic hydrocarbons (like benzo[a]pyrene) contained in tobacco smoke. These adducts preferentially induce G to T transversions in the nontranscribed strand (corresponding to C→A transversion on the other strand) at mCpG sequences. In fact, a mutation signature enriched with C→A mutations has been observed in several tobacco smoking-associated tumors [49]. Previous studies based on mutations in the *TP53* gene have shown that CpG methylation strongly enhances activated benzo[a]pyrene adduct formation [50,51], suggesting that CpG dinucleotides represent preferential targets for exogenous chemical carcinogens. Additional pathways are likely responsible for the link between methylation and somatic mutations we observed, and it is also possible that the mutations directly or indirectly induce methylation changes [52]. Further study is required to explore these mechanisms, which may suggest strategies to delay or reduce the somatic mutation burden.

### Subclonal Mutations and Specific Mutation Signatures Are Associated with Clinical Outcomes

The majority of mutations we identified were subclonal. However, there was large variability across subjects in the fraction of lung tumors’ subclonal mutations. Subclonal mutations were less represented in known driver genes and tended to increase with cancer stage. Notably, APOBEC-related mutations were strongly enriched in subclonal mutations.

We found a striking association between TNSM, specific mutation signatures, and propensity to develop metastasis after the initial lung cancer diagnosis. The risk for metastasis was associated with both synonymous and nonsynonymous mutations and with clonal and subclonal mutations; notably, it also involved new genes not identified as drivers in the TCGA set. This implies that additional adenocarcinomas should be sequenced to identify novel driver genes involved in tumor progression and generate a comprehensive mutation catalog. Interestingly, while the transition mutations C→T and A→G were positively associated with risk of metastasis, the smoking-related C→A mutations and overall transversion ratio were inversely associated. Moreover, like APOBEC-related signatures, C→T transitions were also more frequent in subclonal mutations [49]. Previous studies exploring lung cancer intratumor heterogeneity [53] or the timing of mutational processes [30] suggested that smoking-related mutations were more clonal, early events in lung cancer evolution, while APOBEC-related mutations were late events. Another study showed that lung tumor subclonal mutations were higher in patients (*n* = 3) with relapsed disease [54]. Subclonal mutations were also reported to be associated with reduced survival for leukemia [22]. Based on these observations, mutations developed through endogenous processes—such as C→T mutations because of spontaneous deamination and/or inefficient repair (e.g., TDG-related) and APOBEC-mediated mutations—appear to impact tumor progression through subclonal branching or risk of metastasis, while mutations related to exogenous processes—like C→A and other transversion mutations—alter and dominate the tumor landscape mostly at the beginning of tumor evolution but do not substantially contribute to tumor progression. Larger integrative studies across stage groups and using paired primary–metastatic tumor samples are required to further dissect the evolutionary features of lung cancer.

### Strengths and Limitations

Our primary analyses were conducted within the EAGLE study. Subjects’ selection criteria and modality for enrollment; sample collection and storage; in-person interview for epidemiological variables; clinical follow-up of all lung cancer cases; pathology evaluation; quality control; data management; and laboratory and statistical analyses were all conducted within a single study, following the same standard operating procedures consistently for all subjects. Lung cancer cases were all consecutively enrolled from a set of modern hospitals examining over 80% of the patients in the study catchment area within the Lombardy region of Italy, where over 9 million people are served by a network of health services with universal coverage. These rigorous procedures ensure that sources of bias are limited and allow the assembly of consistent and reproducible results representative of all lung adenocarcinoma cases.

Our study had several limitations. First, our study had a small sample size, which limited the statistical power for studying associations in subgroups of clinical or genomic features. Second, the analysis relied on single tumor samples to investigate the subclonality of somatic mutations and thus could not examine intratumor heterogeneity. Third, not all tumor samples had transcriptome sequencing data because some tumor samples had a limited amount of good quality RNA.

In conclusion, this multidimensional analysis of genomic, clonal evolution, and clinical characteristics of lung adenocarcinoma revealed novel driver genes, epigenome and genome relationships, and specific links between mutation signatures and clinical outcomes. These data may serve as a foundation for development of more effective forms of treatment for lung adenocarcinoma.

## Supporting Information

S1 Fig Copy number alteration (CNA) segmentation, purity estimate, and subclonality analysis. (A) Log R ratio (LRR) and B allele frequency (BAF) data for one tumor sample, profiled using Illumina OmniExpress SNP array. Segmentation was based on BAF using Nexus. Copy number status (LOH, deletion, and amplification) was determined by comparing the sequencing read depth of the tumor DNA to the matched germline DNA, adjusting for total sequencing read depth. Red segments represent deletions; green segments represent amplifications; blue segments represent LOH. (B) For SNP probes in each deletion or LOH, we made a histogram of BAFs and estimated two peaks (μ_1_,μ_2_) using the expectation–maximization (EM) algorithm. The proportion (π) of cells carrying the CNA was estimated based on (μ_1_,μ_2_). After estimating π for all deletions and LOHs, we estimated the density of π using nonparametric statistical methods. Each peak represents one clone. The rightmost clone was determined to be the primary clone, and others were determined as subclones. (C) Orange: subclonal deletions; red: clonal deletions; blue: clonal LOH; purple: subclonal LOH.
(TIF)

S2 Fig A statistical testing framework for determining whether a point mutation was clonal or subclonal after adjusting for CNA status and subclonality status. (A) In the mixed DNA, the tumor (denoted as T) DNA accounts for ***α*** proportion, and the germline DNA (denoted as N) accounts for **1** − ***α*** proportion. The germline genotype is “AA.” Under the null hypothesis that the mutation A→B is clonal, the point mutation should happen before the CNA (deletion) event. We assume that in the tumor DNA, ***β*** proportion of tumor cells have a hemizygous deletion (wild-type allele A is deleted). Then, the mutant allele fraction is calculated as **2**/(**2** − ***αβ***). (B) The calculation of the fraction of mutant allele when ***β*** proportion of tumor cells has a LOH event (and the mutant allele B is duplicated). (C) The distribution of *p*-values for testing whether a point mutation is clonal. A small *p*-value supports that the mutation is subclonal. The left panel is for mutations located in LOH regions. The middle panel is for mutations located in genomic regions without CNA events. The right panel is for mutations in genomic regions with hemizygous deletions.
(TIF)

S3 Fig The figures show the significant genomic regions (FDR q-value <0.05) with focal deletions or amplifications. The figures were produced by GISTIC 2.0.
(TIF)

S4 Fig An experimentally validated gene fusion between *FOXK2* and *KRT20*. (TIF)

S5 Fig An experimentally validated gene fusion between *FOXN1* and *BLMH*. (TIF)

S6 Fig An experimentally validated gene fusion between *RUNX1* and *FARS2* located on two different chromosomes. (TIF)

S1 Table All point somatic mutations identified in the EAGLE study. (XLS)

S2 Table Associations between somatic mutation signatures and smoking, stage, and presurgery chemotherapy. (XLS)

S3 Table Somatic mutations in *POU4F2*, *ZKSCAN1*, and *ASEF* in TCGA, EAGLE, and Broad Institute studies. (XLS)

S4 Table Significant regions with copy number alterations, identified by GISTIC 2.0. (XLSX)

S5 Table Fusion events identified in the EAGLE study. (XLS)

S1 Text Estimate tumor purity using B allele frequency information in SNP arrays. (DOCX)

S2 Text A statistical approach for determining whether a mutation is clonal or subclonal. (DOCX)

S3 Text ASEF protein structure and the mutations with functional relevance. (DOC)

## Abbreviations

BAF: B allele frequency
CGI: CpG island
CNA: copy number aberration
COPD: chronic obstructive pulmonary disease
EAGLE: Environment And Genetics in Lung cancer Etiology
EGF: epidermal growth factor
EM: expectation–maximization
FDR: false discovery rate
FSM: fraction of subclonal mutations
FT: fraction of transversions
GATK: Genome Analysis Toolkit
KRAB: Kruppel-associated box
LeR: leucine-rich region
LOH: loss of heterozygosity
LUAD: lung adenocarcinoma
MAF: mutation allele fraction
MBD4: methyl-CpG binding domain protein
MEGS: mutually exclusive gene set
NMD: nonsense-mediated decay
PGM: Personal Genome Machine
POU4F2: POU class 4 homeobox 2
RIN: RNA Integrity Number
SNV: single nucleotide variant
TDG: thymine DNA glycosylase
TNSM: total number of somatic mutations
WES: whole-exome sequencing
