# Supplementary figures and images for: Somatic Genomics and Clinical Features of Lung Adenocarcinoma: A Retrospective Study
Source: PLoS Med. 2016 Dec 6;13(12):e1002162. doi: 10.1371/journal.pmed.1002162 (PMC5140047; doi:10.1371/journal.pmed.1002162)

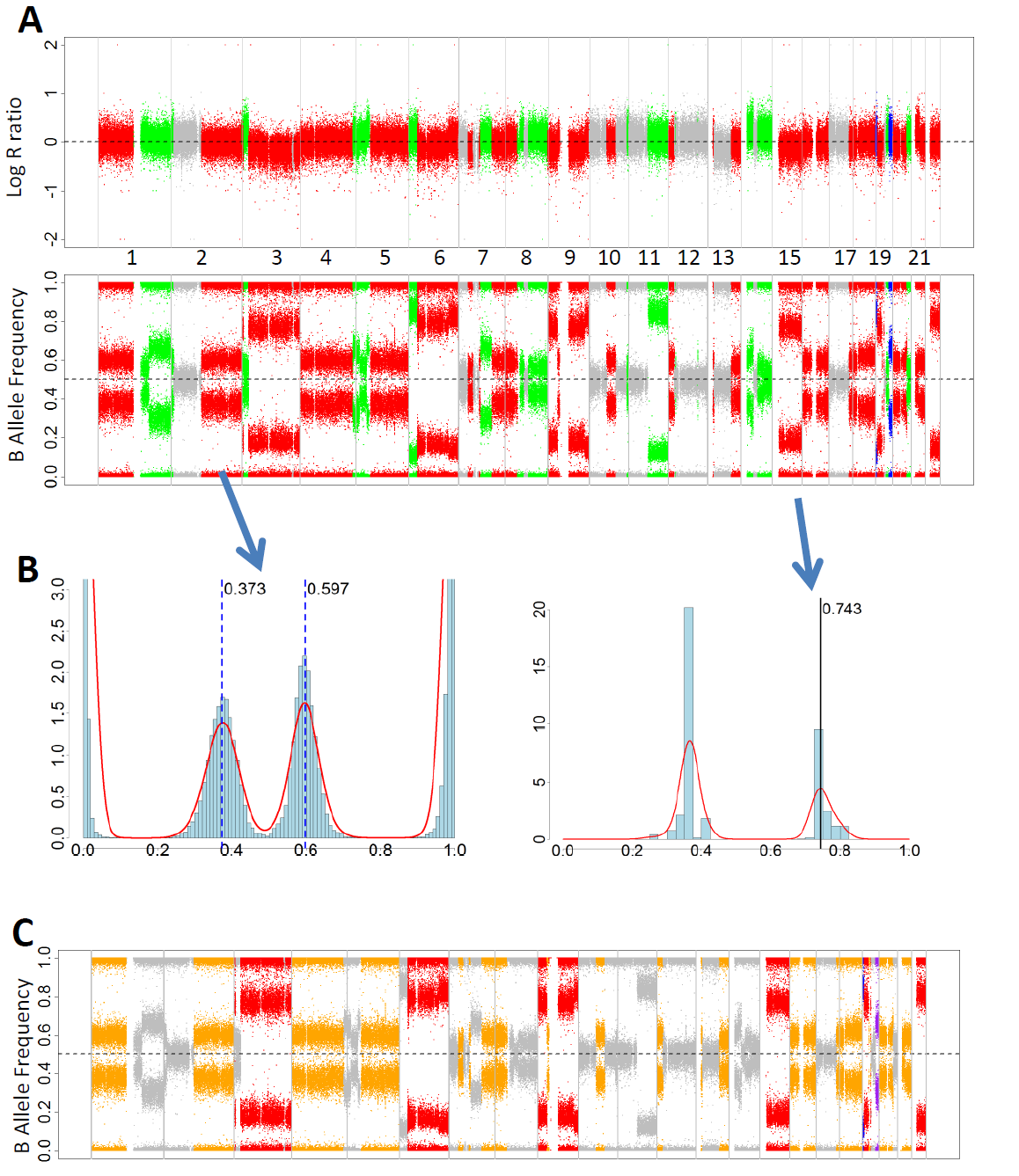

Supplement: S1 Fig — (A) Log R ratio (LRR) and B allele frequency (BAF) data for one tumor sample, profiled using Illumina OmniExpress SNP array. Segmentation was based on BAF using Nexus. Copy number status (LOH, deletion, and amplification) was determined by comparing the sequencing read depth of the tumor DNA to the matched germline DNA, adjusting for total sequencing read depth. Red segments represent deletions; green segments represent amplifications; blue segments represent LOH. (B) For SNP probes in each deletion or LOH, we made a histogram of BAFs and estimated two peaks (μ1,μ2) using the expectation–maximization (EM) algorithm. The proportion (π) of cells carrying the CNA was estimated based on (μ1,μ2). After estimating π for all deletions and LOHs, we estimated the density of π using nonparametric statistical methods. Each peak represents one clone. The rightmost clone was determined to be the primary clone, and others were determined as subclones. (C) Orange: subclonal deletions; red: clonal deletions; blue: clonal LOH; purple: subclonal LOH. (TIF) [file pmed.1002162.s001.tif]

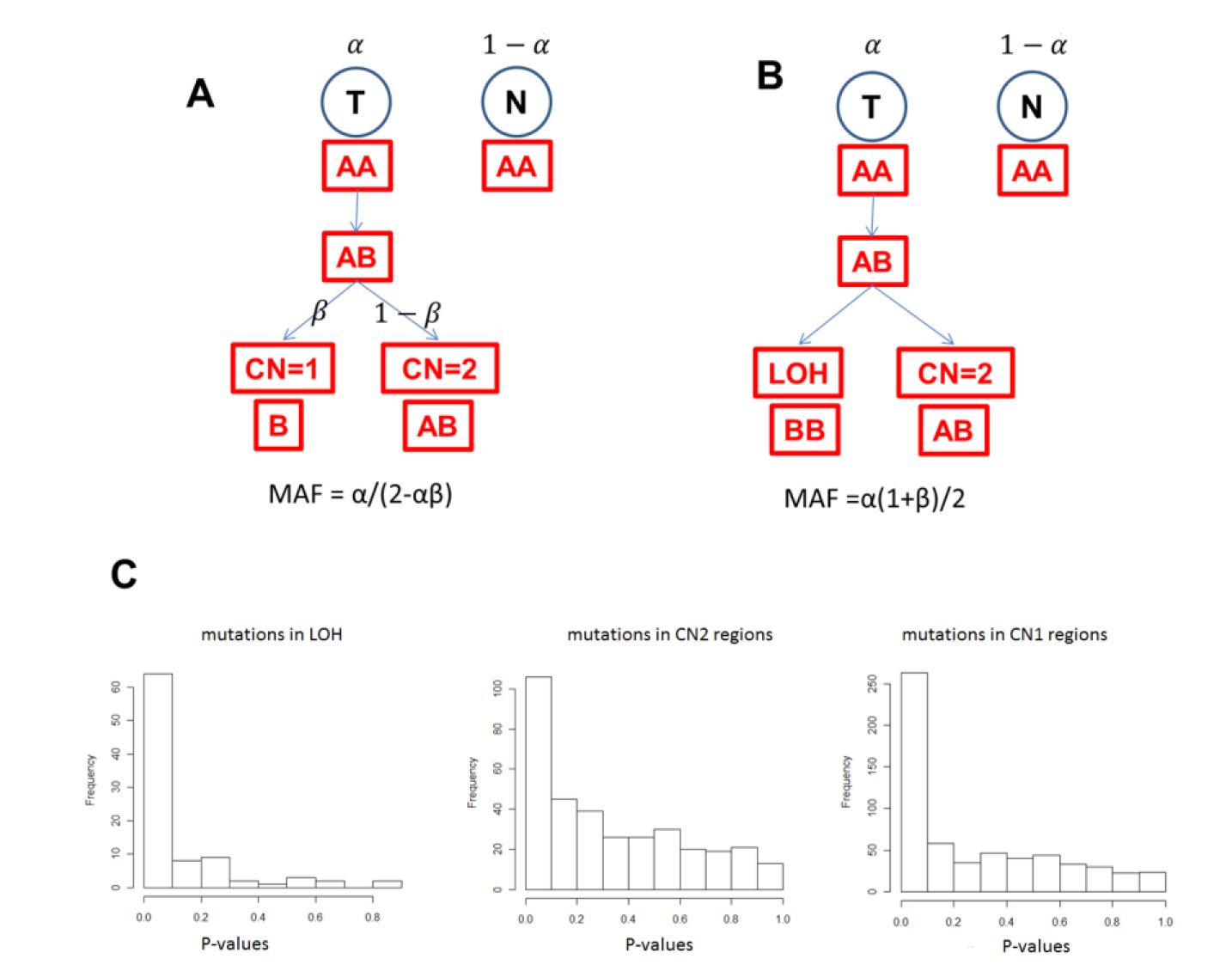

Supplement: S2 Fig — (A) In the mixed DNA, the tumor (denoted as T) DNA accounts for α proportion, and the germline DNA (denoted as N) accounts for 1 − α proportion. The germline genotype is “AA.” Under the null hypothesis that the mutation A→B is clonal, the point mutation should happen before the CNA (deletion) event. We assume that in the tumor DNA, β proportion of tumor cells have a hemizygous deletion (wild-type allele A is deleted). Then, the mutant allele fraction is calculated as 2/(2 − αβ). (B) The calculation of the fraction of mutant allele when β proportion of tumor cells has a LOH event (and the mutant allele B is duplicated). (C) The distribution of p-values for testing whether a point mutation is clonal. A small p-value supports that the mutation is subclonal. The left panel is for mutations located in LOH regions. The middle panel is for mutations located in genomic regions without CNA events. The right panel is for mutations in genomic regions with hemizygous deletions. (TIF) [file pmed.1002162.s002.tif]

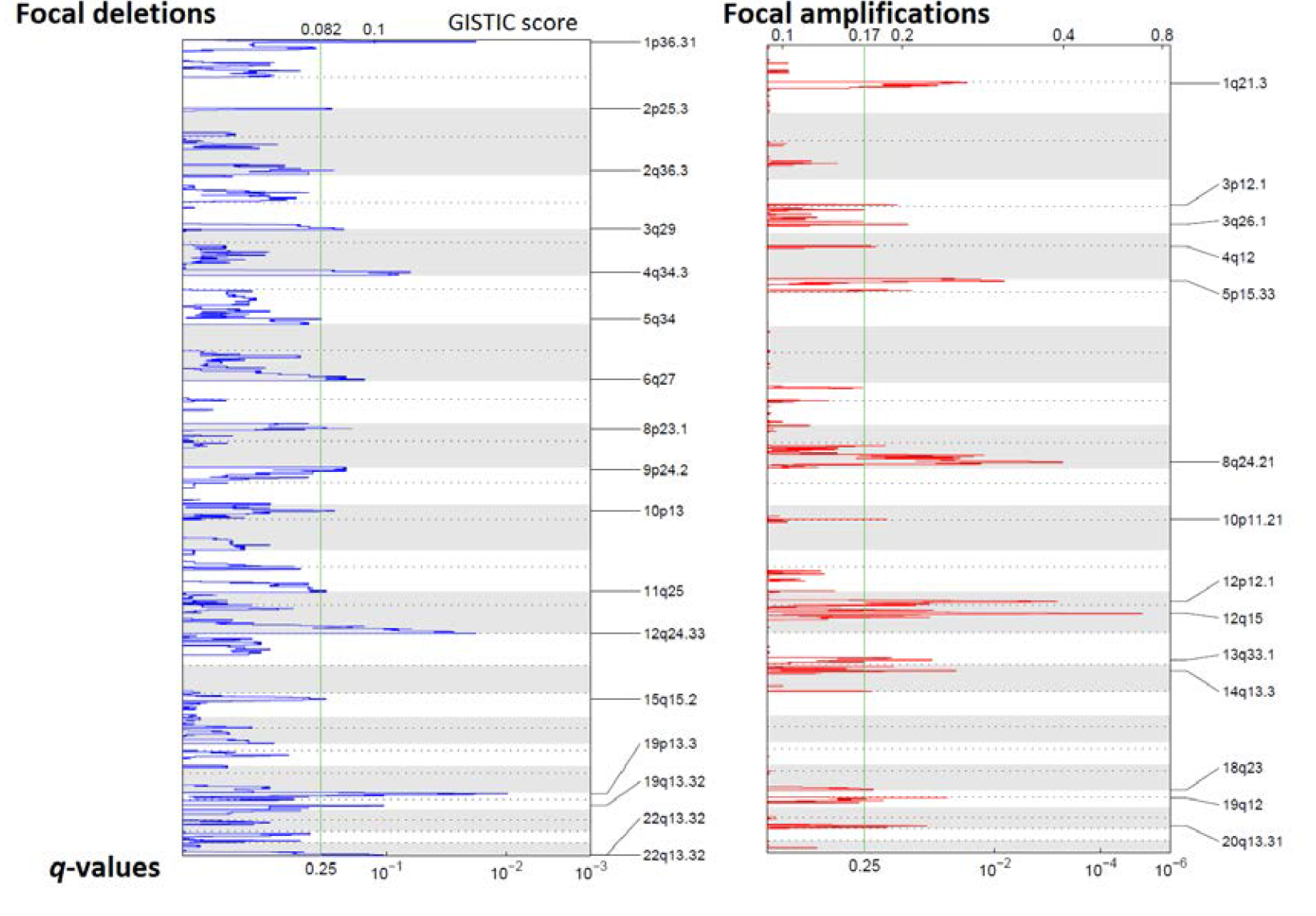

Supplement: S3 Fig — The figures were produced by GISTIC 2.0. (TIF) [file pmed.1002162.s003.tif]

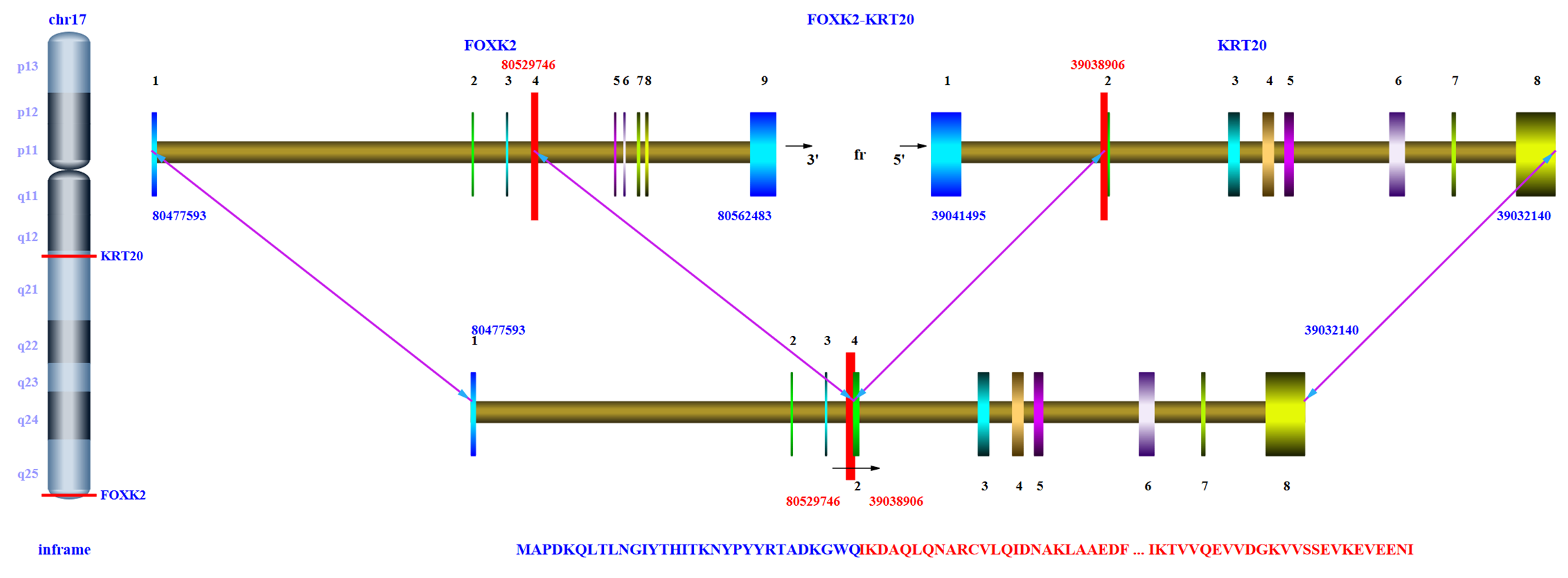

Supplement: S4 Fig — (TIF) [file pmed.1002162.s004.tif]

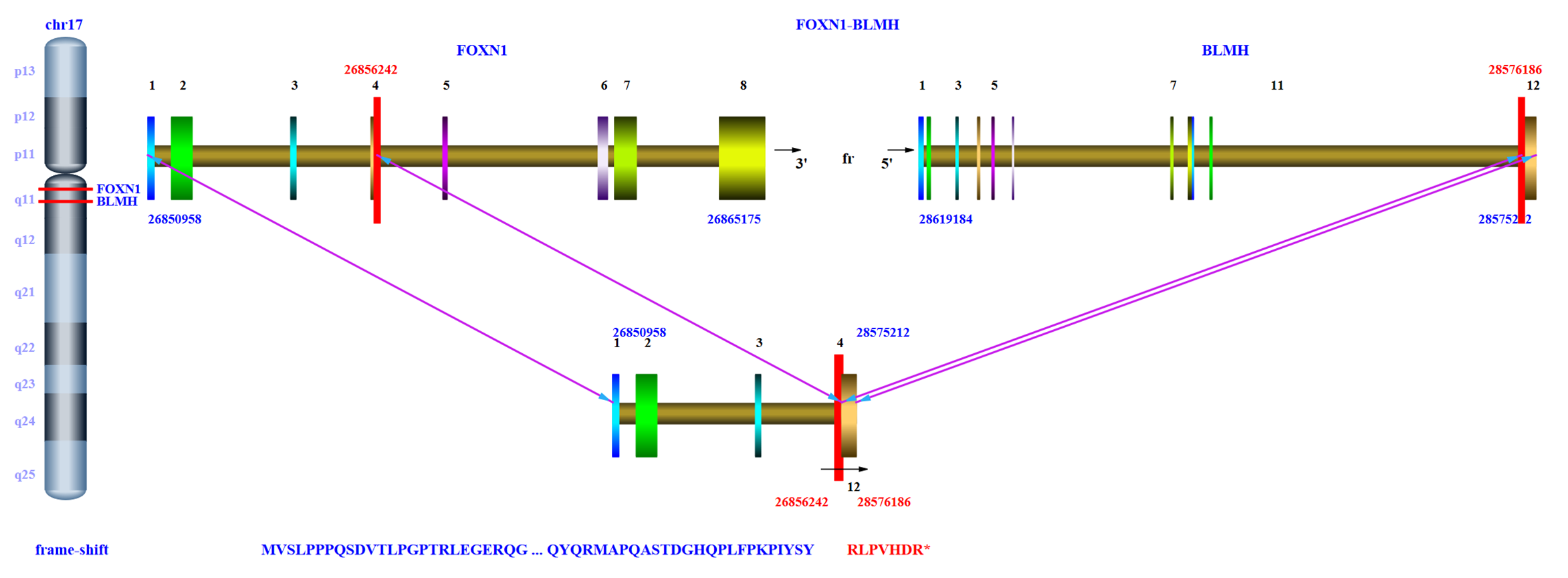

Supplement: S5 Fig — (TIF) [file pmed.1002162.s005.tif]

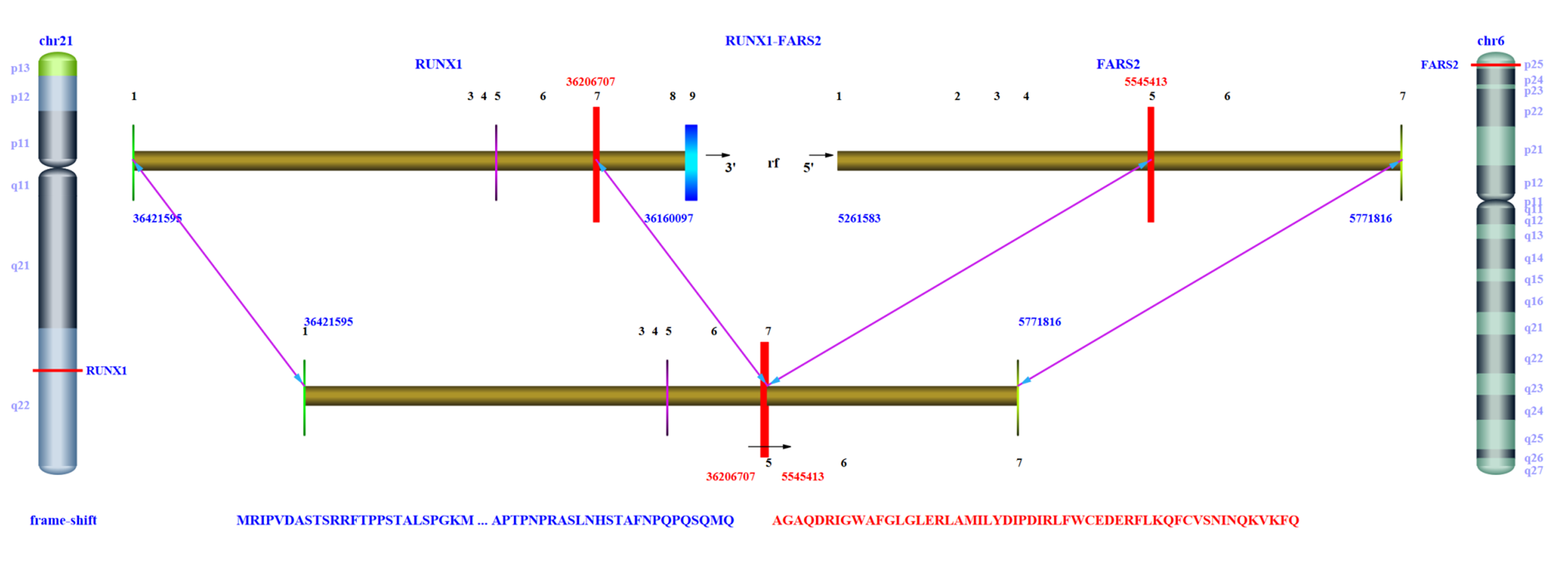

Supplement: S6 Fig — (TIF) [file pmed.1002162.s006.tif]
