# Supplementary material for: Somatic Genomics and Clinical Features of Lung Adenocarcinoma: A Retrospective Study
Source: PLoS Med. 2016 Dec 6;13(12):e1002162. doi: 10.1371/journal.pmed.1002162 (PMC5140047; doi:10.1371/journal.pmed.1002162)
Supplement: S1 Text — (DOCX) [file pmed.1002162.s012.docx]

# Estimate tumor purity using B-allele frequency (BAF) information in SNP arrays

# We use two steps to estimate purity and determine whether a copy number alternation (CNA) was clonal or subclonal.

# Step 1: For each segment mixed by CN2 (copy neutral) and a CNA event (CN1, LOH or CN3), we used the BAF pattern to estimate $\boldsymbol{p}$, the fraction of cells carrying the CNA. First, we made a histogram of BAF and estimated the center of the two BAF bands: $\boldsymbol{\mu}_{\boldsymbol{1}}\boldsymbol{<0.5<}\boldsymbol{\mu}_{\boldsymbol{2}}$. Then, we can estimate $\boldsymbol{p}$ as a function of $\boldsymbol{\mu}_{\boldsymbol{2}}\boldsymbol{-}\boldsymbol{\mu}_{\boldsymbol{1}}$ according to the following table. For CN0, however, BAF pattern is similar to CN2 and thus we cannot estimate $\boldsymbol{p}$. In practice, we found it difficult to decide the absolute copy number for amplifications. Misspecification of the absolute copy number typically severely biases the estimate of $\boldsymbol{p}$ for amplifications. Thus, we only estimate $\boldsymbol{p}$ for CN1 deletions and LOH events. See the left panel of Figure S1B.

|  | $\boldsymbol{\mu}_{\boldsymbol{1}}$ | $\boldsymbol{\mu}_{\boldsymbol{2}}$ | Estimate $\boldsymbol{p}$ |
| --- | --- | --- | --- |
| $\boldsymbol{(1-p)}$ CN2 + $\boldsymbol{p}$CN1 | $\frac{\boldsymbol{1}}{\boldsymbol{2}}\boldsymbol{-}\frac{\boldsymbol{p}}{\boldsymbol{2(2-p)}}$ | $\frac{\boldsymbol{1}}{\boldsymbol{2}}\boldsymbol{+}\frac{\boldsymbol{p}}{\boldsymbol{2(2-p)}}$ | $\frac{\boldsymbol{2(}\boldsymbol{\mu}_{\boldsymbol{2}}\boldsymbol{-}\boldsymbol{\mu}_{\boldsymbol{1}}\boldsymbol{)}}{\boldsymbol{1+(}\boldsymbol{\mu}_{\boldsymbol{2}}\boldsymbol{-}\boldsymbol{\mu}_{\boldsymbol{1}}\boldsymbol{)}}$ |
| $\boldsymbol{(1-p)}$ CN2 + $\boldsymbol{p}$CN3 | $\frac{\boldsymbol{1}}{\boldsymbol{2}}\boldsymbol{-}\frac{\boldsymbol{p}}{\boldsymbol{2(2+p)}}$ | $\frac{\boldsymbol{1}}{\boldsymbol{2}}\boldsymbol{+}\frac{\boldsymbol{p}}{\boldsymbol{2(2+p)}}$ | $\frac{\boldsymbol{2(}\boldsymbol{\mu}_{\boldsymbol{2}}\boldsymbol{-}\boldsymbol{\mu}_{\boldsymbol{1}}\boldsymbol{)}}{\boldsymbol{1-(}\boldsymbol{\mu}_{\boldsymbol{2}}\boldsymbol{-}\boldsymbol{\mu}_{\boldsymbol{1}}\boldsymbol{)}}$ |
| $\boldsymbol{(1-p)}$ CN2 + $\boldsymbol{p}$LOH | $\frac{\boldsymbol{1}}{\boldsymbol{2}}\boldsymbol{-}\frac{\boldsymbol{p}}{\boldsymbol{2}}$ | $\frac{\boldsymbol{1}}{\boldsymbol{2}}\boldsymbol{+}\frac{\boldsymbol{p}}{\boldsymbol{2}}$ | $\boldsymbol{\mu}_{\boldsymbol{2}}\boldsymbol{-}\boldsymbol{\mu}_{\boldsymbol{1}}$ |

# Jacobs et al., Detectable clonal mosaicism and its relationship to aging and cancer. Nat Genet. (2012) 44(6):651-658.

# Step 2: Estimate the number of subclones. For each sample, we derive $\boldsymbol{p}$ for all CN1 deletions and LOH events. After estimating $\boldsymbol{p}$ for all deletions and LOHs, we estimated the density of $\boldsymbol{p}$ using a nonparametric statistical method. Each peak based on the histogram was determined as a subclone. The proportion of cells for each subclone was estimated as the center of each cluster. We assumed that the most right clone was the primary clone and its estimated $\boldsymbol{p}$ represented the purity of the tumor. All CN1 deletions and LOH events with $\boldsymbol{p}$ belonging to the primary clone were determined as clonal CNA. All other CN1 deletions and LOHs were determined as subclonal CNAs.
