# Supplementary material for: Somatic Genomics and Clinical Features of Lung Adenocarcinoma: A Retrospective Study
Source: PLoS Med. 2016 Dec 6;13(12):e1002162. doi: 10.1371/journal.pmed.1002162 (PMC5140047; doi:10.1371/journal.pmed.1002162)
Supplement: S2 Text — (DOCX) [file pmed.1002162.s013.docx]

# A statistical approach for determining whether a mutation is clonal or subclonal

# The copy number alternation (CNA) subclonality was estimated based on B-allele frequency (BAF) information (see S1 Text). Here, we aim to determine whether a SNV is clonal or subclonal using the mutant allele fraction by accounting for tumor purity, CNA and its subclonality. We assume that the number of sequence reads covering the SNV is $\boldsymbol{n}$ and the number of reads with the mutant allele is $\boldsymbol{n}_{\boldsymbol{0}}$. Again, when a SNV is at a locus with amplification, we did not try to determine the subclonality because of the difficulty of estimating the absolute copy number of the amplification.

# We first consider a SNV at a locus with copy number 2. We assume tumor purity $\boldsymbol{\alpha}$. The null hypothesis (*H_0_*) is that the SNV is clonal, e.g., carried by all tumor cells. Under $\boldsymbol{H}_{\boldsymbol{0}}\boldsymbol{,}$ the fraction of sequence reads with the mutant allele is derived as $\boldsymbol{f=\alpha/2}$. The P-value is calculated as $\boldsymbol{P(n\geq}\boldsymbol{n}_{\boldsymbol{0}}\boldsymbol{)}$ with $\boldsymbol{n}_{\boldsymbol{0}}\boldsymbol{\sim Binomial(n,\alpha/2)}$ under $\boldsymbol{H}_{\boldsymbol{0}}$. A small P-value suggests that the SNV is subclonal.

# Second, we consider the scenario that the SNV is at a locus with hemizygous (CN1) deletion. We assume that tumor purity is $\boldsymbol{\alpha}$ and that the CN1 deletion accounted for $\boldsymbol{\beta}$ proportion of tumor cells. Here, $\boldsymbol{\beta}$ was estimated for each CN1 segment in S1 Text. The CNA is subclonal if $\boldsymbol{\beta}\boldsymbol{<}1$. We first derive the expected mutant allele fraction under $\boldsymbol{H}_{\boldsymbol{0}}$ that the mutant allele is carried by all tumor cells (S2A Fig). For $\boldsymbol{\beta}$ proportion of tumor cells with CN1, the mutant allele is carried by the undeleted chromosome; for $\boldsymbol{1-\beta}$ proportion of tumor cells with CN2, the mutant allele is carried by one of the two chromosomes. Then, the expected fraction of mutant allele is given by $\boldsymbol{f=\alpha/(2-\alpha\beta)}$. Thus, the P-value is calculated as $\boldsymbol{P(n\geq}\boldsymbol{n}_{\boldsymbol{0}}\boldsymbol{)}$ with $\boldsymbol{n}_{\boldsymbol{0}}\boldsymbol{\sim Binomial(n,\alpha/(2-\alpha\beta))}$ under $\boldsymbol{H}_{\boldsymbol{0}}$. Note that accounting for subclonality of CN1 has high impact for inference. As a numeric example, if purity $\boldsymbol{\alpha=0.5}$, then $\boldsymbol{f=1/3}$ if $\boldsymbol{\beta=1}$and $\boldsymbol{=0.256}$ if $\boldsymbol{\beta=0.1}$.

# Third, we consider the scenario that the SNV is at a locus with LOH event. We assume that the $\boldsymbol{\beta}$ proportion of tumor cells have LOH. We make a further assumption that the SNV happens before the LOH event. Under this assumption, we can derive that $\boldsymbol{f=\alpha(1+\beta)/2}$ and we can calculate the P-value accordingly.

# S2C Fig gives the distribution of the P-values for testing the clonality of SNV for these three scenarios in one sample. We classified SNVs with P<0.1 as subclonal and SNVs with P>0.1 as clonal. For this sample, about 1/3~1/2 of SNVs were subclonal.
