# Supplementary material for: Somatic Genomics and Clinical Features of Lung Adenocarcinoma: A Retrospective Study
Source: PLoS Med. 2016 Dec 6;13(12):e1002162. doi: 10.1371/journal.pmed.1002162 (PMC5140047; doi:10.1371/journal.pmed.1002162)
Supplement: S3 Text — (DOC) [file pmed.1002162.s014.doc]

**ASEF protein structure and the mutations with functional relevance**

The APC-stimulated guanine nucleotide exchange factor (*ASEF*) gene, also known as Rho guanine nucleotide exchange factor (GEF) 4 (*ARHGEF4*), is a regulator of the cytoskeleton and cell migration [1] and is known for its role in *APC* biology. In *APC* (Min/+) mouse models, which harbor a heterozygous *APC* mutation, ASEF deficiency significantly reduces the numbers of spontaneous tumors [2]. *ASEF* in complex with APC is reportedly pro-angiogenic in this model, and also contributes to an invasive phenotype through the transactivation of MMP-9 downstream of c-Jun amino-terminal kinase (JNK) [2]. In addition to a N-terminal APC-binding region (ABR), ASEF contains a Src-homology-3 (SH3) domain followed by the Dbl-homology (DH) and pleckstrin-homology (PH) domains characteristic of Dbl-family GEFs, and specifically activates GTPase CDC42 [3]. In the resting state, ASEF is present in an auto-inhibited inactive conformation with intra-domain interactions between DH-PH and SH3, which hide the Rac binding domain, DH-PH. Recognition between APC (in its truncated [4] or wild-type form [3]) and ASEF results in the relief of autoinhibition of ASEF and the recruitment of Rac or CDC42, with resulting decrease of cell-cell adhesion, and promotion of cell migration. The 3D structural data is available for ASEF SH3-DH-PH domains in both the auto-inhibited configuration and also as a complex with APC [5], but not for the N-terminal domain, ABR. Among the *ASEF* mutations identified in this study, several have the potential to have an impact on function. For example, G179X (TCGA) is predicted to truncate the protein so that the critical SH3, DH and PH domains needed for auto-inhibition are lost; R249W (EAGLE) and Q433H (EAGLE), part of the SH3 and DH domains, respectively, could impact the ASEF auto-inhibited structure by placing ASEF in an open, auto-active structure ready for interaction with Rac or CDC42; G129V (TCGA) and G141V (EAGLE), which are uncommon substitutions in homologous proteins (BLOSUM62 matrix score) and both lie between the ABR and SH3 domains, and thus could impact the loop region and hence ASEF function; and V424E (TCGA) and C428S (TCGA), both part of the DH domain: the variations result in charged (V424E) and polar (C428S) residue substitutions that could destabilize the structural fold causing protein malfunction (Fig 2).

**References:**

1. Akiyama T, Kawasaki Y. Wnt signalling and the actin cytoskeleton. Oncogene. 2006;25(57):7538-44. doi: 10.1038/sj.onc.1210063. PubMed PMID: WOS:000242514900012.

2. Kawasaki Y, Tsuji S, Muroya K, Furukawa S, Shibata Y, Okuno M, et al. The adenomatous polyposis coli-associated exchange factors Asef and Asef2 are required for adenoma formation in Apc(Min/+)mice. EMBO Rep. 2009;10(12):1355-62. doi: 10.1038/embor.2009.233. PubMed PMID: 19893577; PubMed Central PMCID: PMCPMC2799213.

3. Mitin N, Betts L, Yohe ME, Der CJ, Sondek J, Rossman KL. Release of autoinhibition of ASEF by APC leads to CDC42 activation and tumor suppression. Nat Struct Mol Biol. 2007;14(9):814-23. doi: 10.1038/nsmb1290. PubMed PMID: 17704816; PubMed Central PMCID: PMCPMC2716141.

4. Kawasaki Y, Sato R, Akiyama T. Mutated APC and Asef are involved in the migration of colorectal tumour cells. Nat Cell Biol. 2003;5(3):211-5. PubMed PMID: WOS:000181322500013.

5. Zhang Z, Yang J, Kong EH, Chao WC, Morris EP, da Fonseca PC, et al. Recombinant expression, reconstitution and structure of human anaphase-promoting complex (APC/C). Biochem J. 2013;449(2):365-71. doi: 10.1042/BJ20121374. PubMed PMID: 23078409.
